# Antimicrobial Resistance, Virulence Genes, and Biofilm Formation Capacity Among *Enterococcus species* From Yaks in Aba Tibetan Autonomous Prefecture, China

**DOI:** 10.3389/fmicb.2020.01250

**Published:** 2020-06-12

**Authors:** Pengfei Cui, Lan Feng, Lan Zhang, Juan He, Tianwu An, Xue Fu, Cui Li, Xiaodong Zhao, Yaru Zhai, Hao Li, Wenjun Yan, Huade Li, Xiaolin Luo, Changwei Lei, Hongning Wang, Xin Yang

**Affiliations:** ^1^Animal Disease Prevention and Food Safety Key Laboratory of Sichuan Province, Key Laboratory of Bio-Resources and Eco-Environment, Ministry of Education, College of Life Sciences, Sichuan University, Chengdu, China; ^2^Sichuan Academy of Grassland Sciences, Chengdu, China

**Keywords:** *Enterococcus*, yaks, antibiotic resistance, virulence, biofilm

## Abstract

Yaks provide necessities such as meat and milk for Tibetans living at high altitudes on and around the Qinghai-Tibetan Plateau. Enterococci are ubiquitous members of the animal gut microbiota that can cause biofilm-associated opportunistic infections. Meanwhile, multidrug-resistant *Enterococcus* also poses a serious threat to public health. This study aims to characterize antibiotic resistance, virulence genes, and biofilm formation of enterococci from yaks. From April 2018 to July 2019, we collected 395 fecal samples of yaks in Aba Tibetan Autonomous Prefecture, China. Enterococci isolated from the samples were identified and classified according to the 16S rDNA sequence. The antibiotic resistance of each isolate was detected according to the Kirby-Bauer disk diffusion method, and antibiotic resistance genes were detected by polymerase chain reaction (PCR) and sequencing. Enterococcal biofilms were assessed using standard procedures. Different virulence genes were detected by PCR and sequencing. In total, 381 enterococci strains were recovered, with *Enterococcus faecalis* (41.99%) and *Enterococcus faecium* (37.80%) being the predominant species. Many isolates were multidrug- resistant (60.37%) and showed a high resistance rate to rifampicin (64.30%) and tetracycline (61.54%). We also detected various antimicrobial resistance (AMR) genes in the tested strains. The *E. faecalis* strains had higher frequency of biofilm formation and virulence genes than other enterococcal species. This is the first report that shows yaks are repositories for drug-resistant enterococci with virulent determinants and biofilms that may spread into humans and to environment. This study also provides useful data suggesting that enterococci may pose a potential health risk to yaks. Therefore, active surveillance of AMR and pathogenesis in enterococci from yaks is urgently warranted.

## Introduction

The yaks (*Bos grunniens*) are part of the genus *Bos* within the family Bovidae. As an iconic symbol of high altitude and of Tibet, more than 14 million yaks reside in high-altitude environments of China, Nepal, India, Kyrgyzstan, Pakistan, Russian Federation and Mongolia, mainly across the Qinghai-Tibet Plateau (above 2500–6000 m) in China (Mi et al., 2013). Yaks are essential animals for Tibetans and other nomadic pastoralists in high-altitude environments owing to their provision of the basic necessities (such as milk, meat, transportation, hides for tented accommodation and dung for fuel) (Qiu et al., 2015). However, frequent outbreaks of bacterial diseases in yaks cause serious economic losses and healthy concern to local people (Bandyopadhyay et al., 2012).

Enterococci are the members of Gram-positive lactic acid-producing bacteria widely distributed in the gut microbiota of humans and other animals (García-Solache and Rice, 2019). Enterococci belong to facultative anaerobes and are highly tolerant to diverse environmental conditions, such as extreme PH, salt concentrations and a wide range of temperature (from 10 to >45°C) (Arias and Murray, 2012). The genus *Enterococcus* includes *Enterococcus faecalis*, *Enterococcus faecium, Enterococcus hirae, Enterococcus mundtii*, and *Enterococcus casseliflavus*, etc. (Ahmed and Baptiste, 2018). Both *E. faecium* and *E. faecalis* are the predominant species that are easily isolated from feces of healthy organisms (Landete et al., 2018). Commensal enterococci are generally not pathogenic in healthy hosts; however, in susceptible hosts, they can cause infections in the wounds, dysbiotic gastrointestinal tract, urinary tract, and endocarditis. More importantly, these infections are associated with biofilms (Ch’ng et al., 2019). Enterococcal biofilms increase their hardiness and contribute to both persistence during infection and contamination of the food industry and the environment (Piggot et al., 2012; Da-Silva-Fernandes et al., 2017). The ability of enterococci to form biofilms also increases their intrinsic tolerance to antibiotics and thus is a serious obstacle for the treatment of infections (Ch’ng et al., 2019).

Global public health is severely threatened by antimicrobial resistance (AMR), the major source of which is composed of the “ESKAPE” pathogens (*E. faecium*, *Staphylococcus aureus*, *Klebsiella* spp., *Acinetobacter baumannii*, *Pseudomonas aeruginosa*, and *Enterobacter* spp.) (Woksepp et al., 2014). Among them, *E. faecium* is considered as a typical multidrug-resistant (MDR) pathogen owing to both its enormous capacity to develop acquired resistance during antimicrobials chemotherapy and its intrinsic resistance to various antibiotics such as aminoglycosides and β-lactam-based antibiotics (Dale et al., 2015). The World Health Organization (WHO) recently released a list of drug-resistant “priority pathogens” that pose a significant threat to public health and urgently need new therapies (World Health Organization [WHO], 2017). Vancomycin-resistant *E. faecium* is classified as a Priority 2 pathogen in this list. Other enterococcal species that frequently occur in animals, such as *E. durans*, *E. hirae*, and *E. casseliflavus*, are also appropriate indicators of AMR in Gram-positive bacteria and their appearance can constitute a ‘pool’ of resistant genes, which may be transferred either to other commensal bacteria or even to pathogenic bacteria (Hamed et al., 2018).

As enterococci potentially transfer resistant genes from enteric bacteria in animals to humans via the food chain and they are potential pathogens, they have become a global public health concern. In previous reports, available data showed MDR *Enterococcus* with a variety of virulent determinants isolated from commensal animals, such as chickens, pigs and cattle (Diarra et al., 2010; Beshiru et al., 2017; De Jong et al., 2019; Igbinosa and Beshiru, 2019). However, there has been no available data on enterococci isolated from yaks. During the sampling process, we consulted local Tibetans and teachers of the Sichuan Academy of Grassland Sciences about the use of antibiotics in yaks. Some Tibetans used tetracycline antibiotics and cephalosporins, many Tibetans did not know the specific drug used to treat yak diseases. The level of antibiotic resistance and virulence determinants in enterococci isolated from yaks can reflect the selection pressure as a result of the use of antimicrobials and the potential pathogenesis, respectively.

Some enterococcal infections are associated with the production of biofilms, which increase enterococci’s intrinsic resistance to antimicrobials. Also, enterococci have the ability to horizontally transfer antibiotic resistance genes in biofilms at high rates, and the transfer is promoted by Epa, Ebp, PrgABC (Bhatty et al., 2015; Dale et al., 2015; La-Rosa et al., 2016). Both antibiotic resistance and biofilms of enterococci can increase the difficulty to treat bacterial diseases in yaks. It is therefore important to monitor AMR, virulence genes, and biofilm formation capacity of enterococci in yaks. In this study, we provided the first comprehensive analysis of antibiotic resistance, virulence genes and biofilm formation of *Enterococcus* species isolated from yaks.

## Materials and Methods

### Sample Collection

From April 2018 to July 2019, 395 non-repeated fecal samples were collected from healthy yaks from 37 different farms in the Aba TAP Tibetan Autonomous Prefecture (Sichuan), Sichuan Province, China. During the sampling process, we used sterile swabs to collect fresh feces, and then placed them into 10 mL aseptic tubes. We placed the collected samples with ice packs and later shipped them to the laboratory within 24 h of collection for isolating bacteria.

### Isolation and Identification of *Enterococcus* Species

At the stage of pre-enrichment, we placed the samples into 10 mL aseptic tubes containing 4 mL BHI (Brain Heart Infusion) broth, before incubating them at 37°C for 18–24 h with a rotation speed of 200 rpm. The enriched cultures were inoculated to Bile Esculin Azide agar (*Enterococcus* selective agar). One colony was selected for each sample. Subsequently, we used the BD Phoenix-100 Automated Microbiology System (BD Dignostic System, Sparks, MD, United States) to identify all the isolated strains. The confirmed isolates were stored in 25% glycerol containing BHI broth at −80°C. According to the instructions of the TIANamp Column Bacteria Genomic DNA Purification Kit (Beijing Tiangen Biotech, Beijing, China), total bacterial DNA was extracted from all the *Enterococcus* strains for PCR templates. The genomic DNA solution was stored at −20°C.

To identify the enterococcal species, all the isolates were screened for the presence of 16S rDNA gene by PCR. The PCR products were then directly sent to a sequencing company (Chengdu Sangon Biological Engineering Technology & Services, Co., Ltd.) and DNA sequences were analyzed online using BLAST^1^.

### Antimicrobial Sensitivity Test

The antibiotic sensitivity profile of all enterococci was determined according to Clinical and Laboratory Standards Institute [CLSI] (2017) guidelines. The following antimicrobials (all purchased from Oxoid, Thermo Fisher Scientific, Basingstoke, United Kingdom) were used: ampicillin (AMP, 10 μg), penicillin (PEN, 10 units), erythromycin (ERY, 15 μg), tetracycline (TET, 30 μg), rifampicin (RIF, 5 μg), ciprofloxacin (CIP, 5 μg), chloramphenicol (CHL, 30 μg), vancomycin (VAN, 30 μg), linezolid (LZD, 30 μg), fosfomycin (FOS, 50 μg) and nitrofurantoin (NIT, 300 μg). *E. faecalis* ATCC 29212 was used as a quality control strain.

### Detection of Antimicrobial Resistance Genes

The emergence of drug-resistant genes associated with tetracycline (*tet*A, *tet*B, *tet*M, *tet*L), erythromycin (*erm*A, *erm*B), ciprofloxacin (*qnr*A, *qnr*B, *qnr*S, *qep*A), linezolid (*optr*A, *poxt*A), chloramphenicol (*cat*) and vancomycin (*van*A, *van*B) was determined by PCR using specific primers (Supplementary Table S1). Positive PCR products were sequenced by Chengdu Sangon Biological Engineering Technology & Services, Co., Ltd.

### Biofilm Formation

Biofilm assays were performed according to the guidance of a method (Stepanović et al., 2007). First, the purified *Enterococcus* colonies were resuspended in 10 mL of Tryptic Soy Broth (TSB) supplemented with 1% glucose, incubated at 37°C for 18–20 h. Then, for each strain tested, 20 μl of bacterial suspensions were transferred to three wells of sterile 96-well polystyrene microtiter plates containing 180 μl of TSB supplemented with 1% glucose. *E. faecalis* (ATCC 29212) was used as the positive control, and 200 μl of broths (TSB with 1% glucose) were used as the negative control. The microtiter plates were incubated for 24 h at 37°C, washed with sterile phosphate-buffered solution (PBS), dried at 28 ± 2°C, and stained with crystal violet for 30 min. The wells were washed twice with sterile deionized water and dried. Crystal violet dye bound to adherent cells was resuspended in 150 mL of 99% ethanol. The OD readings from respective wells were determined at 570 nm. Each assay was determined three times. The formation of biofilm was classified as negative, weak, moderate, or strong. The cut-off value (ODc) was defined as the mean OD value above three standard deviations (SD) of the negative control: ODc = average OD of negative control + (3 × SD of negative control). Each *Enterococcus* isolate was classified as follows: OD < ODc = non-biofilm producers (category 0); ODc < OD < 2ODc = weak biofilm producers (category 1); 2ODc < OD < 4ODc = moderate biofilm producers (category 2); and OD > 4ODc = strong biofilm producers (category 3).

### Screening for Virulence Associated Genes

Specific primers (Supplementary Table S2) were used for PCR testing of all *Enterococcus* isolates to detect virulence genes (selected according to their functional characteristics, including secreted factors and cell surface determination cluster) encoding *cyl*A, *gel*E, *agg*, *ace*, *ebp*A, *ebp*B, *ebp*C, *efa*A, *hyl*, and *srt*A. Appropriate virulence genes were used as positive controls and sterile water as negative controls in all tests.

### Statistical Analysis

All statistical analyses were performed using GraphPad Prism 8. Fisher’s exact test for samples were used. *P* < 0.05 was considered statistically significant.

## Results

### Sample Collection and the Species of Enterococci

In this study, we collected 395 non-duplicated fresh samples in 37 farms. Out of the samples, a collection of 381 enterococci were identified. Hence, the isolation rate of *Enterococcus* isolates was 96.46% (381**/**395) in the current investigation. The number of isolated strains of all enterococcal species is shown in Table 1, and the most frequent *Enterococcus* spp. was *E. faecalis* (*n* = 160), followed by *E. faecium* (*n* = 144).

**TABLE 1:** Numbers of isolates of enterococcal species from yaks in Aba TAP (from April 2018 to July 2019).

Species	Isolates	Percentage of rate
*E. faecalis*	160	41.99
*E. faecium*	144	37.80
*E. hirae*	37	9.71
*E. mundtii*	29	7.61
*E. durans*	6	1.58
*E. casseliflavus*	3	0.79
*E. avium*	1	0.26
*E. gallinarum*	1	0.26

Due to the different scales of the farms, the number of enterococci isolated in each farm varies. On most farms, *E. faecalis* and *E. faecium* are the main isolates. However, we isolated 8 *E. mundtii* strains (accounting for 50%) on farm 3. Moreover, six different kinds of *Enterococcus* species were detected on farm 5. The details of the isolation of enterococci on each farm are shown in Supplementary Table S3.

### Antimicrobial Sensitivity Test

Table 2 shows antibiotic non-susceptible (intermediate and resistant) *Enterococcus* species isolated from yaks, which exhibit high rates of resistance to RIF (64.30%) and TET (61.54%), with rates of resistance to ERY (35.96%), CIP (35.96%), CHL (26.25%), NIT (24.93%), PEN (17.32%), and AMP (15.22%). However, 10.76, 3.94, and 2.10% of strains were resistant to LZD, VAN, and FOS, respectively. Of the isolates, 60.37% (*n* = 230) were multi-drug resistant (MDR, resistant to at least three different classes of antibiotics). The various resistance patterns were observed, of which TET-ERY-RIF was more common.

**TABLE 2:** Distribution of antibiotic non-susceptible *Enterococcus* species isolated from yaks.

Antimicrobial	*E. faecalis* *N* = 160	*E. faecium* *N* = 144	*E. hirae* *N* = 37	*E. mundtii* *N* = 29	*E. durans* *N* = 6	*E. casseliflavus* *N* = 3	*E. gallinarum* *N* = 1	*E. avium* *N* = 1	No. (%) of isolate Total no.
RIF	148	134	28	1	3	2	1	1	318 (83.46%)
TET	135	103	14	4	2	1	1	1	261 (68.50%)
CIP	139	137	30	7	2	1	0	1	317 (83.20%)
ERY	155	129	23	21	5	2	1	1	337 (88.45%)
CHL	106	72	7	0	0	0	0	0	185 (48.56%)
NIT	46	112	18	6	3	1	0	1	187 (49.08%)
PEN	3	57	3	2	0	0	0	1	66 (17.32%)
AMP	1	53	1	2	0	0	0	1	58 (15.22%)
LZD	102	100	26	4	5	1	1	1	240 (62.99%)
VAN	146	48	4	11	3	0	1	1	318 (83.46%)
FOS	16	35	2	3	2	1	0	0	59 (15.49%)

We further analyzed the relationship between resistance phenotypes and enterococcal species. We found that *E. faecium* was the most antibiotic resistant species, with a multi-drug resistance rate of 75.69%, followed by *E. faecalis*, whose multi-drug resistance rate was 68.13%. Whereas most of *E. mundtii* isolates were sensitive to antibiotics, only two strains were MDR. Our data showed that *E. faecium* was more resistant to AMP, PEN, and NIT than *E. faecalis* (*P* < 0.0001; Fisher’s exact test). Moreover, we found that erythromycin and tetracycline had a combined resistance trend, with 137 erythromycin-resistant isolates and 133 *Enterococcus* strains resistant to tetracycline together.

### Detection of Antimicrobial Resistance Genes

Table 3 shows the detection of 15 AMR genes. The highest rate of resistance gene was *tet*M gene, which accounted for 54.07% of the strains. Other tetracycline resistances *tet*A, *tet*B, and *tet*L were detected in 6.56, 2.62, and 45.14% of the strains, respectively. The erythromycin resistance gene *erm*B was found in 30.45% of the isolates, and *erm*A gene was only presented in 15.75%. The *cat* gene responsible for chloramphenicol resistance, was detected in 25.98% of the strains. Moreover, the oxazolidinone resistance genes (*optr*A, *poxt*A) were found in 3.94 and 0.26% of the isolates, respectively. We didn’t detect ciprofloxacin resistance genes (*qn*rA, *qnr*B, *qnr*S, and *qep*A) in ciprofloxacin resistant strains. The vancomycin resistance genes *van*A and *van*B were also not detected in this study.

**TABLE 3:** Prevalence and distribution antimicrobial resistance genes in *Enterococcus* isolates recovered from yaks.

Resistance genes	*E. faecalis* *N* = 160	*E. faecium* *N* = 144	*E. hirae* *N* = 37	*E. mundtii* *N* = 29	*E. durans* *N* = 6	*E. casseliflavus* *N* = 3	*E. gallinarum* *N* = 1	*E. avium* *N* = 1	No. (%) of isolate Total no.
*erm*A	29	30	1	0	0	0	0	0	60 (15.75%)
*erm*B	73	37	5	0	0	0	1	0	116 (30.45%)
*tet*A	10	13	0	0	0	0	1	1	25 (6.56%)
*tet*B	0	9	0	0	1	0	0	0	10 (2.62%)
*tet*M	116	78	8	2	1	0	0	1	206 (54.07%)
*tet*L	95	67	6	2	1	0	0	1	172 (45.14%)
*cat*	77	22	0	0	0	0	0	0	99 (25.98%)
*qnrA*	0	0	0	0	0	0	0	0	0
*qnr*B	0	0	0	0	0	0	0	0	0
*qnr*S	0	0	0	0	0	0	0	0	0
*qep*A	0	0	0	0	0	0	0	0	0
*optr*A	10	5	0	0	0	0	0	0	15 (3.94%)
*poxt*A	0	1	0	0	0	0	0	0	1 (0.26%)
*van*A	0	0	0	0	0	0	0	0	0
*van*B	0	0	0	0	0	0	0	0	0

The phenotypic and genotypic patterns of the AMR detected in the isolates are shown in Supplementary Material S4.

### Analyze the Capacity of Biofilm Formation

Biofilm formation of the Enterococci includes the following: non-formers, 124 (32.55%); weak formers, 112 (29.40%); moderate formers, 113 (29.66%); and strong formers, 32 (8.40%). Overall, 257 (67.45%) were biofilm formers. Biofilm formation of the tested *E. faecalis* was statistically significantly higher than that of *E. mundtii* strains (*P* = 0.0089; Fisher’s exact test). Table 4 summarizes the biofilm-forming strength in *Enterococcus* species isolated from yaks.

**TABLE 4:** Association between biofilm-forming strength in TSB Broth with 1% glucose and *Enterococcus* species (no. of strains/%).

Biofilm strength	*E. faecalis* *N* = 160	*E. faecium* *N* = 144	*E. hirae* *N* = 37	*E. mundtii* *N* = 29	*E. durans* *N* = 6	*E. casseliflavus* *N* = 3	*E. gallinarum* *N* = 1	*E. avium* *N* = 1	No. (%) of isolate Total no.
No biofilm	22	63	15	20	2	1	0	1	124 (32.55%)
Weak	43	43	14	9	2	1	0	0	112 (29.40)
Moderate Strong	79 16	26 12	6 2	0 0	2 0	0 1	0 1	0 0	113 (29.66%) 32 (8.40%)

### Prevalence of Virulence Genes in Enterococci

Table 5 shows the prevalence of virulence genes detected in all isolates, as follows: *cyl*A, 73 (19.16%); *gel*E, 160 (41.99%); *agg*, 162 (42.52%); *ace*, 246 (64.57%); *ebp*A, 150 (39.37%); *ebp*B, 61 (16.01%); *ebp*C, 175 (45.93%); *esp*, 122 (32.02%); *efa*A, 300 (78.74%); *hyl*, 143 (37.53%); and *srt*A, 202 (53.02%). Only three *E. faecalis* isolates were simultaneously positive to all tested virulence genes, while 24 *E. mundtii* strains were negative to all tested virulence genes. These results indicate that *E. faecalis* had the potential for higher virulence and *E. mundtii* the potential for lower virulence among enterococcal species.

**TABLE 5:** Prevalence and distribution virulence genes in *Enterococcus* isolates recovered from yaks.

Virulence genes	*E. faecalis* *N* = 160	*E. faecium* *N* = 144	*E. hirae* *N* = 37	*E. mundtii* *N* = 29	*E. durans* *N* = 6	*E. casseliflavus* *N* = 3	*E. gallinarum* *N* = 1	*E. avium* *N* = 1	No. (%) of isolate Total no.
*cyl*A	73	0	0	0	0	0	0	0	73 (19.16%)
*gel*E	123	29	8	0	0	0	0	0	160 (41.99%)
*agg*	115	41	0	0	4	2	0	0	162 (42.52%)
*ace*	156	76	10	1	1	1	1	0	246 (64.57%)
*ebp*A	112	28	9	0	0	1	0	0	150 (39.37%)
*ebp*B	61	0	0	0	0	0	0	0	61 (16.01%)
*ebp*C	126	35	12	0	0	2	0	0	175 (45.93%)
*esp*	78	32	8	0	2	1	1	0	122 (32.02%)
*efa*A	150	137	10	3	0	0	0	0	300 (78.74%)
*hyl*	101	38	0	3	1	0	0	0	143 (37.53%)
*srt*A	150	41	11	0	0	0	0	0	202 (53.02%)

We then analyzed the relationship between virulence genes and biofilm formation, which shows there was a correlation between biofilm formation and the presence of the *ace* gene in *E. faecium* (*P* < 0.0001; Fisher’s exact test). The presence of *gel*E and *cyl*A genes were mainly found in *E. faecalis* (*P* < 0.0001; Fisher’s exact test), and these genes did not have a statistically correlation for the formation of biofilms in *E. faecalis* (*Pgel*E = 0.7348, *Pcyl*A = 0.6894; Fisher’s exact test).

The genotypic patterns of the virulence factors and biofilms detected in the isolates are shown in Supplementary Material S5.

## Discussion

Compared with findings about enterococci of other commensal animals, available data on enterococci isolated from yaks is still inadequate. The yak is vital to the production system of the Tibetans and other nomadic herders in high-altitude regions (Xiao-Yun et al., 2006). Here, we provide the comprehensive analysis of the antimicrobial susceptibility, biofilm formation, and virulence genes of several enterococcal species isolated from yaks. We observed a high proportion of multidrug-resistant enterococci with virulence factors in yaks.

In current study, 381 Enterococci were isolated. Unlike previous studies, the isolation rate of *E. faecalis* was slightly higher than that of *E. faecium* (De Jong et al., 2018, 2019; Lei et al., 2019). Moreover, the isolation rate of *E. mundtii* was 7.61% (*n* = 29), which contradicts the findings from previous studies that show a very low isolation rate of *E. mundtii* in other commensal animals (Kim et al., 2016; De Jong et al., 2018; Osman et al., 2019). These results indicate that *Enterococcus* species composition varies in different host environments.

Enterococcal resistance and tolerance to antimicrobials is a serious threat to global health that needs to be addressed as a priority. Enterococci are intrinsically resistant to various antibiotics (for example, aminoglycosides, clindamycin, and β-lactam-based antibiotics). Due to the low permeability of enterococcal cell wall to large aminoglycoside molecules, enterococci are moderately resistant to aminoglycosides and are more prevalence in *E. faecium* than *E. faecalis* (Aslangul et al., 2006; Abat et al., 2016). Enterococci have the ability to overexpress penicillin-binding proteins with low affinity for β-lactams, which allows enterococci intrinsically resistant to penicillin (Murray, 1997; Duez et al., 2001). Compared with *E. faecalis*, *E. faecium* is more intrinsically resistant to antimicrobials. However, *E. faecalis* can show tolerance to antibiotics by forming a thicker biofilm. Biofilms of *E. faecalis* isolated from clinic show increased tolerance to vancomycin and tigecycline (Hashem et al., 2017). In the present study, we found that 15 *E. faecalis* strains were resistant to vancomycin. However, vancomycin-resistant genes were not detected in these strains. It may be due to the formation of biofilms that enhance tolerance to vancomycin.

Enterococci can readily acquire resistance to antimicrobials during antibiotics chemotherapies. In this study, 60.37% of the *Enterococcus* isolates were MDR. We observed that erythromycin and tetracycline had a combined resistance trend, which was in line with the findings of previous study (Kim et al., 2019). High antibiotic resistance in the study area may be a marker for differences in multiple factors such as antimicrobials use, disease control measures, or genetic mutations leading to multidrug-resistant phenotypes (Jadhav et al., 2011). Our study reveal that local Tibetan people’s inappropriate use of antibiotics caused MDR enterococci to appear in yaks. Furthermore, yak is a free-grazing animal that can move around in a large geographic area, and thus yak feces containing MDR bacteria can also cause pollution to the local ecological environment. Therefore, robust monitoring programs are needed to control the spread of AMR in yaks.

The association among enterococcal species and resistance phenotypes varied in this study. Overall, *E. faecium* strains had a higher rate of MDR, whereas most of *E. mundtii* isolates were sensitive to antibiotics. Similarly, the higher prevalence of MDR *E. faecium* among *Enterococcus* spp. isolated from other animals has been documented previously (Novais et al., 2013; De Jong et al., 2018). Moreover, enterococcal species also had significant differences in antibiotic resistance among various antibiotics, for we observed that *E. faecium* was more resistant to penicillin and ampicillin than *E. faecalis*. This was in line with the findings of other study (Novais et al., 2013). Since enterococci that produce β-lactamase are extremely rare, the acquired resistance of *E. faecium* to ampicillin is mainly due to the penicillin-binding protein5 (*pbp*5) mutation, which has a lower affinity for β-lactam antibiotics, while the fact that *E. faecalis* has acquired resistance to ampicillin is mainly due to *pbp*4 mutation (Rice et al., 2004; Ono et al., 2005). Therefore, our results indicate that *pbp*5 of *E. faecium* may be more susceptible to mutations than *pbp*4 of *E. faecalis* under antibiotic stress.

According to the results of the antibiotic resistance phenotype, various resistance genes were detected in *Enterococcus* strains. Among the tetracycline resistance genes, *tet*M and *tet*L were significantly present in tetracycline-resistant enterococci. Similarly, *erm*B was more than *erm*A present in erythromycin-resistant enterococci. These results were in line with the previous studies (Schwaiger et al., 2009; Kim et al., 2019). We also detected oxazolidinone resistance genes *optr*A and *poxt*A. Oxazolidinones (tedizolid and linezolid) are effective antibiotics for the treatment of multidrug resistant Gram-positive bacterial (including vancomycin-resistant *Enterococcus*) infections, and these resistant genes can usually be linked with mobile genetic elements for horizontal transmission (Hao et al., 2019; Lei et al., 2019), so we should standardize the use of oxazolidinone and strengthen the detection of oxazolidinone-resistant enterococci to prevent the spread of resistant genes to the environment and humans. Enterococci resistance to ciprofloxacin is usually due to the mutations in the *gyr*A and *par*C quinolone resistance-determining region (Onodera et al., 2002; Leavis et al., 2016), so we did not detect plasmid-mediated quinolone resistance genes in isolated strains.

Enterococcal biofilms have been observed in many infections. Moreover, Biofilm is an important source of pollution in the food processing industry and enterococci isolated from food have the ability to form biofilms (Ch’ng et al., 2019; Igbinosa and Beshiru, 2019). Therefore, we determined biofilm formation capacity among *Enterococcus* species from yaks and its correlation with virulence genes. We found that many of the *E. faecalis* isolates showed higher-level biofilm formation and virulence genes, whereas most of the tested *E. mundtii* strains were negative to biofilm and all tested of virulence genes. Our findings corroborate the results reported that *E. faecalis* was the first-most frequent *Enterococcus* species associated with disease (Gawryszewska et al., 2017), while *E. mundtii* was low in virulence and infrequently associated to infections (Repizo et al., 2014).

We found that *ace* had a correlation with biofilm formation in *E. faecium*. Compared with many factors involved in *E. faecalis* biofilm formation, several *E. faecium* genes are involved in the development of biofilm, including *esp*, *ebp*ABC, and *ace* (Sava et al., 2010; Lim et al., 2017). However, we observed the low prevalence of *ebp*A, *ebp*B, *ebp*C, and *esp* genes in *E. faecium*. Hemolysin–cytolysin (Cyl) and the proteases gelatinase (GelE) are important secreted factors and play a key role in enterococcal pathogenesis. These genes (*cyl*A and *gel*E) were more often found in *E. faecalis* strains. Similar findings were also reported that *E. faecalis* more often carried *cyl*A and *gel*E genes among enterococcal species (Golob et al., 2019; Stępień-Pyśniak et al., 2019).

Most biofilm-associated infections are caused by a combination of multiple microorganisms, and usually two or more of species can be found at the site of infection. Enterococci can account for a large proportion of the population in wound infections identified by next generation sequencing technology (Holá et al., 2010; Dworniczek et al., 2012). Isolation of *E. faecalis* is often accompanied by other bacteria. For example, *Proteus mirabilis* was recovered from nearly 40% of *E. faecalis* biofilms (Macleod and Stickler, 2007). It was also found on catheters together with *Klebsiella pneumoniae* and *Escherichia coli* (Galvan et al., 2016). Meanwhile, enterococci can act as gene pool to spread antibiotic resistance within and between species. This may lead to the aggravation of clinical symptoms and increase the difficulty of diagnosis and control of yak diseases. For the local Tibetans, yak milk and meat are not only the main protein food, but also an important economic source by exporting for high-end consumption (Yue et al., 2013), and thus AMR genes of enterococci in yaks can transfer to humans via the food chain. Therefore, it is altogether fitting and proper that we analyze the antibiotic resistance and virulence of enterococci isolated from yaks.

## Conclusion

The results of this study reveal that 60.37% isolated strains were MDR and 9 antibiotic-resistant genes were detected. Likewise, great frequency of biofilm formation and virulence genes were observed among the isolated enterococci. The emergence of AMR genes and virulence genes in enterococci from yaks is a serious concern because they could be transmitted to humans through the food chain and the spread of these genes could significantly limit the treatment options for MDR bacteria. Finally, the study demonstrate that yaks are reservoirs of antimicrobial resistant enterococci with potential virulence. Therefore, we should initiate robust surveillance programs to control and monitor the use of antibiotics.

## Data Availability Statement

All datasets generated for this study are included in the article/Supplementary Material.

## Ethics Statement

The animal study was reviewed and approved by the College of Life Science, Sichuan University affiliation ethics committee, and all efforts were made to minimize animal suffering.

## Author Contributions

PC designed the study and wrote the manuscript. LF, XF, TA, XZ, HuL, and XL collected the samples. PC, JH, LZ, CLi, YZ, HaL, and WY performed the experiments. HW, CLe, and XY supervised the research.

## Conflict of Interest

The authors declare that the research was conducted in the absence of any commercial or financial relationships that could be construed as a potential conflict of interest.

## Footnotes

1 http://blast.ncbi.nlm.nih.gov/
