# Supplementary material for: Antimicrobial Resistance, Virulence Genes, and Biofilm Formation Capacity Among Enterococcus species From Yaks in Aba Tibetan Autonomous Prefecture, China
Source: Front Microbiol. 2020 Jun 12;11:1250. doi: 10.3389/fmicb.2020.01250 (PMC7304059; doi:10.3389/fmicb.2020.01250)
Supplement: Supplementary file 1 [file Data_Sheet_1.zip › Data Sheet 1.docx]

**Table S1:** Primers used for determination of 16S rDNA and antimicrobial resistance genes in current study.

| Genes | Oligonucleotide sequences (5′-3′) | Length (bp) | References |
| --- | --- | --- | --- |
| 16S rDNA | F: AGAGTTTGATCMTGGCTCAG  R: TACGGYTACCTTGTTACGACTT | 1506 | Kim et al., 2012 |
| *tet*A | F: GGCACCGAATGCGTATGAT  R: AAGCGAGCGGGTTGAGAG | 480 | Choi and Woo, 2015 |
| *tet*B | F: CTCAGTATTCCAAGCCTTTC  R: CTAAGCACTTGTCTCCTGTT | 416 | Choi and Woo, 2015 |
| *tet*M | F: GTTAAATAGTGTTCTTGGAG  R: CTAAGATATGGCTCTAACAA | 657 | Choi and Woo, 2015 |
| *tet*L | F: ATAAATTGTTTCGGGTCGGTAAT  R: AACCAGCCAACTAATGACAATGAT | 1077 | Choi and Woo, 2015 |
| *erm*A | F: TCTAAAAAGCATGTAAAAGAA  R: CTTCGATAGTTTATTAATATTAGT | 645 | Sutcliffe et al., 1996 |
| *erm*B | F: GAAAAGGTACTCAACCAAATA  R: AGTAACGGTACTTAAATTGTTTAC | 639 | Sutcliffe et al., 1996 |
| *qnr*A | F: GCCCGCTTCTACAATCAAGT  R: GGCAGCACTATTACTCCCAAG | 347 | Hamed et al., 2018 |
| *qnr*B | F: TATGGCTCTGGCACTCGTT  R: GCATCTTTCAGCATCGCAC | 193 | Hamed et al., 2018 |
| *qnr*S | F: TCGGCACCACAACTTTTCAC  R: TCACACGCACGGAACTCTAT | 255 | Hamed et al., 2018 |
| *qep*A | F: TCTACGGGCTCAAGCAGTTG  R: ACAGCGAACCGATGACGAAG | 312 | Hamed et al., 2018 |
| *cat* | F: GGATATGAAATTTATCCCTC  R: CAATCATCTACCCTATGAAT | 486 | Osman et al., 2019 |
| *optr*A | F: AGGTGGTCAGCGAACTAA  R: ATCAACTGTTCCCATTCA | 1395 | Wang et al., 2015 |
| *poxt*A  *van*A  *van*B | F: GGTGGATTTACCGACACCGT  R: GACCAGTGGAAATGCCCGTA  F: GCGCGGTCCACTTGTAGATA  R: TGAGCAACCCCCAAACAGTA  F: AGACATTCCGGTCGAGGAAC  R: GCTGTCAATTAGTGCGGGAA | 943  314  220 | Lei et al., 2019  Nam et al., 2012  Nam et al., 2012 |

**Table S2:** Primers used for determination of virulence genes in current study.

| Genes | Oligonucleotide sequences (5′-3′) | Length (bp) | References |
| --- | --- | --- | --- |
| *cyl*A | F: ACTCGGGGATTGATAGGC  R: GCTGCTAAAGCTGCGCTT | 688 | Vankerckhoven et al., 2004 |
| *gel*E | F: ACCCCGTATCATTGGTTT  R: ACGCATTGCTTTTCCATC | 419 | Lopes-Mde et al., 2006 |
| *agg* | F: CACGTAATTCTTGCCCACCA  R: AAACGGCAAGACAAGTAAATA | 520 | Seputiene et al., 2012 |
| *ace* | F: CAAGCATTATTGGCAGCGTT  R: TCTATCACATTCGGTTGCG | 320 | Duprè et al., 2003 |
| *ebp*A | F: CCATTTGCAGAAGCAAGAATG  R: GAGTGAAAGTTCCTCCTCTAG | 613 | Hashem et al., 2017 |
| *ebp*B | F: CATTAGCAGAGGCATCGCAA  R: CAAGTGGTGGTAAGTCATAGG | 504 | Hashem et al., 2017 |
| *ebp*C | F: CTGCTACGAATATGGTGGTG  R: GGTGTTTGATTGTTTGCTTC | 487 | Hashem et al., 2017 |
| *esp* | F: CGGTCATACCGACGACCAAA  R: TGTCACATCGCCATCGACTT | 745 | Vankerckhoven et al., 2004 |
| *efa*Afs | F: GACAGACCCTCACGAATA  R: AGTTCATCATGCTGTAGTA | 705 | Eaton and Gasson, 2001 |
| *efa*Afm  *hyl*  *srt*A | F: AACAGATCCGCATGAATA  R: CATTTCATCATCTGATAGTAF  F: ACAGAAGAGCTGCAGGAAATG  R: GACTGACGTCCAAGTTTCCAA  F: GTATCCTTTTGTTAGCGATGC  R: TGTCCTCGAACTAATAACCGA | 735  276  612 | Eaton and Gasson, 2001  Vankerckhoven et al., 20044  Hashem et al., 2017 |

**Table S3:** Numbers of isolates of enterococcal species from yaks on each farm.

| Farm | *E. faecalis* | *E. faecium* | *E. hirae* | *E. mundtii* | *E. durans* | *E. casseliflavus* | *E. gallinarum* | *E avium* | Total no. |
| --- | --- | --- | --- | --- | --- | --- | --- | --- | --- |
|  | N=160 | N=144 | N=37 | N=29 | N=6 | N=3 | N=1 | N=1 |  |
| 1 | 7 | 9 | 2 | 0 | 0 | 0 | 0 | 0 | 18 |
| 2 | 7 | 4 | 0 | 0 | 0 | 0 | 0 | 0 | 11 |
| 3 | 2 | 3 | 3 | 8 | 0 | 0 | 0 | 0 | 16 |
| 4 | 2 | 3 | 1 | 0 | 0 | 0 | 0 | 0 | 6 |
| 5 | 1 | 6 | 3 | 1 | 1 | 1 | 0 | 0 | 13 |
| 6 | 7 | 2 | 0 | 0 | 0 | 0 | 0 | 0 | 9 |
| 7 | 2 | 9 | 1 | 0 | 0 | 0 | 0 | 0 | 12 |
| 8 | 5 | 3 | 0 | 0 | 0 | 0 | 0 | 0 | 8 |
| 9 | 5 | 2 | 1 | 1 | 0 | 0 | 0 | 0 | 9 |
| 10 | 6 | 4 | 0 | 0 | 0 | 0 | 0 | 0 | 10 |
| 11 | 3 | 1 | 0 | 3 | 0 | 1 | 0 | 0 | 8 |
| 12 | 4 | 0 | 0 | 1 | 1 | 0 | 0 | 0 | 6 |
| 13 | 8 | 3 | 3 | 1 | 0 | 0 | 0 | 0 | 15 |
| 14 | 9 | 4 | 0 | 1 | 1 | 0 | 0 | 0 | 15 |
| 15  16  17  18  19  20  21  22  23  24  25  26  27  28  29  30  31  32  33  34  35  36  37 | 3  5  9  3  1  8  2  2  7  4  9  2  4  1  5  3  3  5  0  5  3  5  3 | 1  1  4  5  4  2  3  5  2  6  2  4  1  6  4  5  8  5  7  2  7  3  4 | 0  0  1  1  3  1  0  0  1  0  0  0  3  1  5  1  5  0  0  0  1  0  0 | 2  0  3  1  1  1  1  0  0  0  2  0  0  0  0  0  1  0  0  0  0  1  0 | 0  0  0  0  0  1  0  1  0  0  0  0  1  0  0  0  0  0  0  0  0  0  0 | 0  0  0  0  0  0  0  0  0  0  0  0  0  0  0  1  0  0  0  0  0  0  0 | 0  1  0  0  0  0  0  0  0  0  0  0  0  0  0  0  0  0  0  0  0  0  0 | 0  0  0  0  0  0  0  0  0  0  0  1  0  0  0  0  0  0  0  0  0  0  0 | 6  7  17  10  9  13  6  8  10  10  13  7  9  8  14  10  17  10  7  7  11  9  7 |
